# Patient Interest in the Development of a Center for Ehlers-Danlos Syndrome/Hypermobility Spectrum Disorder in the Chicagoland Region

**DOI:** 10.21203/rs.3.rs-3034682/v1

**Published:** 2023-07-14

**Authors:** Wendy Wagner, Tom Doyle, Clair Francomano, Dacre Knight, Colin Halverson

**Affiliations:** Wendy 4 Therapy; Indiana University School of Medicine; Indiana University School of Medicine; Mayo Clinic’s Campus in Florida; Indiana University-Purdue University Columbus

## Abstract

**Background:**

The Ehlers-Danlos Syndromes (EDS) are a set of connective tissue disorders that are hereditary in nature and characterized by joint hypermobility and tissue fragility. The complex nature of this unique patient population requires multidisciplinary care, but appropriate centers for such care do not exist in large portions of the country. Need for more integrated services has been identified in the Chicagoland region. In order to explore and begin to address barriers to seeking out appropriate care facing EDS patients in this region, we developed an online survey which we circulated through EDS social media groups for Chicagoland patients.

**Results:**

Three hundred and nine unique respondents participated. We found that there exists a strong medical need for and interest in the development of a center in the region, and participants reported that, if made available to them, that they would make extensive and regular use of such a facility.

**Conclusions:**

We conclude that the establishment of a collaborative medical center specializing in the diagnosis and treatment of EDS, HSD, and related disorders in the Chicagoland area would greatly benefit patients by providing comprehensive care, alleviate the burden on overworked healthcare providers, and generate revenue for medical facilities.

## Introduction

The Ehlers-Danlos Syndromes (EDS) are a set of connective tissue disorders that are hereditary in nature and characterized by joint hypermobility and tissue fragility.^1–3^ While musculoskeletal pain and joint laxity are the most common clinical presentations, EDS, being a connective tissue disorder, can affect numerous body systems.^4,5^ Manifestations and secondary impairments associated with EDS differ among individuals in terms of severity and symptoms.^6^ The extent of disability caused by EDS likewise varies, depending on the organs affected and the resulting dysfunction. Common and often equally disabling comorbidities include neurological and immunological disorders, as well as pain, fatigue, and digestive dysfunction experienced by people with EDS.^7^

Recent research indicates that for every 5000 patients, one expects to find 10 diagnosed cases of EDS.^8^ However, previous research has found the case-rate to be much lower, with a prevalence of EDS at 1 in every 5000 births.^1^

Among EDS, hypermobile EDS (hEDS) is the most common type, while other types are much rarer.^7^ hEDS has been referred to as a poorly understood disease that is often difficult to diagnose.^9^ As a result, hEDS frequently goes undiagnosed or is only detected after prolonged delays and significant time spent in the medical system before an accurate diagnosis is made.^10^ Part of the difficulty is that hypermobility itself is relatively common. The prevalence of symptomatic joint hypermobility in a university-aged population has been estimated at 12.5%.^11^ It has also been reported that 50% of patients who present to outpatient orthopedic physical therapy clinics have underlying joint hypermobility regardless of whether it was their primary presenting symptom, compared to 30% in a control group.^12^

It has been suggested that hEDS (a diagnosis confirmed using the 2017 diagnostic criteria) and HSD (a clinical diagnosis given to those who present with symptomatic joint hypermobility but who do not meet the strict criteria of hEDS) may not distinguish two separate patient populations.^13^ The combined prevalence of HSD and hEDS falls somewhere between 1 in 600 to 1 in 900. Expert opinion is that HSD is common and that hEDS is likely to be common; however, at this time, the exact prevalence of each separate diagnostic category has not been clearly established.^14^

Patients with EDS are often misdiagnosed, poorly understood, and inadequately managed by healthcare professionals.^15,16^ There are several common comorbid conditions, including: dysautonomia, postural orthostatic tachycardia syndrome (POTS), mast cell activation syndrome/disorder (MCAS/MCAD), craniocervical instability (CCI), anxiety, depression, migraine, gastrointestinal distress, TMJ, fibromyalgia, and arthritis^17^ and in this paper will be referred to as “related disorders.” Patients with EDS therefore need clinical support from a wide range of medical experts. Our siloed healthcare system tends to fail to link symptoms that can be traced to an underlying connective tissue disorder. It also tends to discount these patients and their presenting symptoms as “rare” and unrelated despite their single origin.^17^

As general clinicians tend to lack knowledge about hEDS and HSD and related disorders,^18^ patients often have difficulty accessing appropriate and collaborative care to address the wide range of symptoms and associated impairments.^10^ This ultimately contributes to a shortage and overburdening of current clinicians and medical practices with expertise in diagnosis and management.^18^ In addition, access to multidisciplinary teams and relevant specialists is often limited or nonexistent in rural areas.^19^ The great majority of university centers lack multidisciplinary teams with expertise in and resources for patients with hEDS and HSD.^13^

The authors have noted anecdotally in their own practice that few specialty EDS or HSD medical providers exist in the Chicagoland area, collaboration among providers rarely occurs, existing specialized practices are overburdened and resultant long wait lists for office visits all prove significant barriers to care for our patients. It is hypothesized that the creation of a specialty center in the Chicago area may begin to address these issues.

Such a center would represent an opportunity for a healthcare system to differentiate from their “competition” by establishing niche programs focused on particular areas of medicine that have a deficit of high-quality and effective medical providers in a particular geographic area. Such a center would deliver care in a comprehensive, interdisciplinary system and is committed to providing diagnosis, patient care, advocacy, research, community and professional education.^20^

To that end, a survey was sent out to members of local hEDS and HSD social media groups and to patients being seen at local clinics. The survey was designed to identify their needs, desires, and barriers to care with an ultimate goal of understanding how a multidisciplinary clinic might be structured to address these issues. This survey explored participants’ current healthcare related to hEDS and HSD and related disorders as well their interest in having access to a center in the Chicagoland area in the future.

## Research Methods and Design

### Ethical Approval

The study was approved by the Institutional Review Board of Indiana University (#17881). Written consent document was obtained before the survey was completed.

### Study Design and Recruitment

This research project was designed to capture information about past and anticipated future healthcare needs of the Chicagoland EDS/HSD population. In order to do that, a review of the relevant literature was conducted and a survey was developed using the online tool SurveyMonkey^™^. We then shared a link with people who have been diagnosed with or suspect they have EDS/HSD and related disorders. These participants were recruited from members of the “Chicago EDS Support Group” Facebook group and the “EDS Illinois” Facebook group. The link was also shared with multiple medical providers in the Chicagoland area who are dedicated to treating this unique population to be shared with their patients. The survey was open for approximately one month, during which time two reminder emails were sent and social media posts were posted. At the end of one month, the survey was closed and data were analyzed.

### Survey Instrument and Analysis

The survey was designed to capture data regarding: 1) patient demographics, 2) past and current health care needs, access to services, experiences and satisfaction with services, 3) anticipated future healthcare needs and expression of future needs of such of services, 4) level of interest and willingness to access proposed collaborative healthcare services in the future. For some questions, the option was given to the participant to select “other” and write in their response in a comment box should the provided answers not adequately reflect their desired response. The survey is provided in the Supplementary Online Materials.

A pilot of the survey was conducted with five participants to confirm readability and flow. Survey responses were not collected anonymously; however, once data analysis and reporting were complete, all participants were deidentified. Descriptive statistics were calculated to estimate the responses to the different questions.

## Results

### Demographics:

A total of 309 unique respondents participated in the survey. The majority identified as female (89%) and were between 25 and 44 years old (53%). Sixty-eight percent had a formal diagnosis of hEDS or HSD, with an additional 20% having a suspected EDS/HSD diagnosis. The remainder had a formal or suspected diagnosis of some other form of EDS. Twenty-two percent lived within the city limits of Chicago, and 62% lived in the Chicagoland suburbs. Seventy-four percent had private insurance. Other demographic information is found in Table 1.

### Health Status and Healthcare Utilization:

Nearly all participants (94%) said that their symptoms negatively affected their ability to work, go to school and/or participate in preferred recreational activities. Respondents were asked whether they had been diagnosed with any of ten of the most common comorbidities for the population.^17^ Participants reported an average of 6.6 of the 10 conditions as either formally diagnosed or suspected. The greatest number of participants indicated dysautonomia (78%), anxiety (69%), migraines (67%), mast cell activation syndrome (66%), irritable bowel syndrome (IBS) (65%), and depression (58%). The full breakdown of these ten diagnoses is found in Table 2.

Seventy-four percent of our respondents had private insurance. Despite this, 30% said they had spent $10,000 or more the preceding calendar year on healthcare expenses, including insurance premiums, copays, deductible, out of pocket, medications/supplements, medical equipment with 9% of all respondents reporting that they spent more than $20,000. Further breakdown of these statistics is found in Table 3.

Over the last five years, the majority (51%) had accessed care at Northwestern Memorial Hospital. This was followed by Northshore (39%), Advocate Health (32%), and Rush University (29%). (Participants were allowed to select as many medical centers as they had accessed, and the average participant had accessed care at 3.5 different hospital systems within the relevant time period.) However, participants felt that the current clinical facilities in the Chicagoland area were insufficient in their ability to address participants healthcare needs. As one participant stated, “The need is great-many doctors in the Chicago area are completely unfamiliar with EDS” Another stated that “Chicago is an EDS physician desert.”

Forty-six percent had seen 11 or more medical providers in pursuit of care for their EDS/HSD, with 28% accessing 16 or more providers. Within that time period, the majority had sought care with primary care (86%), physical therapy (78%), neurology (68%), cardiology (65%), and rheumatology (64%), gastroenterology (60%), and psychology (66%). A full breakdown of the various medical disciplines from which they sought care can be found in the Appendix.

### Satisfaction with Current Care:

Nearly half (49%) of our respondents were dissatisfied or very dissatisfied with their current local medical support for their EDS or HSD symptoms. Only 5% reported that they were “very satisfied” with their current care. The majority (82%) found it somewhat difficult, difficult, or very difficult to access quality care for their condition. Nearly all participants (99%) believed that their providers’ ability to collaborate on their care was important to their health outcomes. (Seventy-five percent said it was “extremely important.”) Yet, the majority (63%) expressed dissatisfaction with the collaboration of their past or current providers, with 22% stating they were “very dissatisfied.” A lack of understanding of EDS and HSD was seen as a critical barrier to getting appropriate care. One participant commented, “I have been searching in earnest statewide for clinicians of just about any kind who focus on, research, or are at least familiar enough with EDS.”

Participants were asked the longest amount of time they had waited for a diagnosis of EDS/HSD or of a related disorder; the average was 17.2 years.

Nearly all (95%) of respondents said they had suffered from medical trauma or medical gaslighting, which we defined in the survey as “the experience a patient has when a medical provider discounts or minimizes symptoms or inappropriately considers them psychosocial instead of physical in origin.” The majority (56%) said that their experiences with medical trauma and/or medical gaslighting led them to “avoid or put off” medical care, and an additional 11% said that they went so far as to avoid seeking medical care “as much as possible” due to these experiences. One respondent explained their experience as follows: “I had been told repeatedly that nothing was wrong with me for several years. I have been mocked, belittled, ignored, and discriminated against by providers and peers.”

### Future Goals for Accessing Care

If a collaborative system of expert care in the form of an EDS/HSD center were available to them in the Chicagoland area, nearly all respondents (95%) said that they would utilize it within the next one to two years. In fact, 82% said that they were “very likely” to do so. Only 2% said that they were unlikely to make use of such a center.

One participant provided a representative summary of the respondents’ attitudes: “My biggest problem is being tossed around from practice to practice, each assuming that my condition falls under another specialty. I need a place where it is believed that EDS belongs in their care.” Another participant expressed exhaustion from the pressures the current system places on individual patients: “It’s become practically a full time job to be my own advocate and to coordinate my care. And it’s exhausting. A clinic that could coordinate my care and see my symptoms and whole body together and not in isolated silos would be life-changing.” Lack of education and lack of communication between physicians were regularly noted as barriers to appropriate care: “With complex patients, more should be done about doctors working together to resolve symptoms versus the patient being passed back and forth trying to coordinate a care plan as this is very exhausting for patients.” One participant concluded: “I would jump at the chance to have knowledgeable doctors all in one place.”

## Discussion

The respondents were primarily young adult females living in the Chicago suburbs. They spend a significant amount of their financial resources on healthcare, have experienced a long delay in diagnosis and treatment, are generally dissatisfied with their current healthcare and find it difficult to access services related to the symptoms that are most disabling for them. They have experienced medical gaslighting to a degree that it impairs their ability to access the care they need and have verbalized a lived experience where the symptoms that they experience are significantly impacting their ability to work/go to school and/or participate in preferred recreational activities. Although they believe that collaboration between their healthcare providers would greatly improve their health outcomes, they have not experienced such collaboration. If a collaborative healthcare clinic dedicated to treating patients with EDS, HSD, and related conditions was developed, they were very likely to access such services in the future.

As patients in the Chicagoland area report past dissatisfaction with services and poor access to qualified services, it appears that the creation of a center dedicated to treating patients with EDS/HSD and related disorders would be highly beneficial, valued and accessed. With the many specialty visits, diagnostic testing and treatment interventions that this population accesses across the medical system, there appears to be a benefit to both the wellness of the Chicagoland patient population and to the Chicago medical system(s) that would set up such a system to serve them.

Recent findings have suggested that the use of complementary and alternative medical care (CAM) is very common among the EDS population, in part due to a perceived failure of conventional methods in managing chronic pain and other symptoms.^21^ In our survey, 169 (55%) respondents reported accessing CAM or integrative care in the last five years, and 231 (90%) said they were either somewhat or very likely to make use of such services at a center within the next two years. Moreover, there was very high utilization (78%) and interest in physical therapy (92%). A well-designed center should therefore consider including services beyond those associated with conventional biomedical specialties.

Research has also documented limited knowledge about EDS/HSD among health care providers; who often lack experience with these disorders and express a discomfort with diagnosing them.^18^ Patients report inappropriate assessments and inaccurate diagnoses, and many develop a mistrust of healthcare providers and negative expectations for future healthcare encounters, which may lead them to avoid further medical consultations.^15,22^ In general, access to comprehensive, multidisciplinary care teams with expertise in EDS/HSD is lacking in terms of both geography and appropriate education.^10,23^ Finally, many healthcare professionals have inaccurate expectations that a diagnostic genetic test will be readily available for every heritable disorder of connective tissue, despite no such test being available for hEDS or any form of HSD.^24^

As a result of the lack of medical providers trained in the unique needs of these complex patients, current medical practices dedicated to treating these populations are overburdened and have significant waitlists and delays for office visits. Current healthcare providers risk burnout and withdrawal from care for this community. As these disorders affect multiple body systems and as the associated symptoms overlap and even affect each other, “[n]o individual healthcare professional can truly manage all aspects of these conditions at an expert level.”^14^

Centers for patients with EDS, HSD, and related disorders are limited. Other EDS clinics that the authors have been involved with in the United States have reported that new EDS patients are typically a younger population with commercial insurance: At the EDS/HSD program at Mayo Clinic in Jacksonville, Florida, 93% of their 411 unique patients had commercial insurance.^25^ This has resulted in higher reimbursement rates for the facility. They also report that for each new EDS patient referred from outside, 86% were referred for additional specialty consults, 33% completed imaging, and 69% completed non-lab testing.^25^

These facts further reinforce the hypothesis that establishing a center for this patient population would be mutually beneficial not only to the patient population but also for the institution that would implement such a center. Major medical centers in Chicago have centers for other medical conditions already established as well as significant infrastructure already in place to support them.

### Limitations:

There are some limitations of this study. Participants self-reported whether they had been diagnosed with EDS or HSD; diagnostic history was not further verified. There is likely a higher response rate among physical therapy patients as recruitment was partially accomplished through the professional network of one of the authors (WW), who is a physical therapist.

### Implications and Future Research:

Our results show that there is dissatisfaction with current care and a significant need for improved future care for the EDS/HSD population. It is therefore critical to identify resources to better support them in the future and to establish necessary resources where they do not currently exist. It has been proposed that patients with diagnoses of EDS/HSD and related disorders would benefit from access to a medical system that provides them with: (a) one single point of entry into coordinated healthcare services, (b) a team of EDS/HSD-informed providers who are sensitive to the connection between their symptoms, (c) access to a network of EDS/HSD-aware specialty medical disciplines for referral and consultation, and (d) access a collaborative medical model in which providers work together to discuss and plan treatment for these complex patients. It is anticipated that early identification and diagnosis of EDS/HSD will enable: (a) education of the patient and supporting family, (b) reduction of secondary impairments, (c) avoidance of unnecessary and sometimes harmful interventions, and (d) the provision of appropriate care.

In designing such a center, it is important first to solicit feedback from the local EDS/HSD community in order to identify preferred features and potential barriers to access including: cost, distance willing to travel, locations of providers, patient flow through the system, coordination of care among providers, timing of appointments, reporting, etcetera. For continued quality improvement, it would also be appropriate to follow a population of patients longitudinally, as they access such a system. This would allow administrators of the center to modify the program in a participatory and evidence-based manner, and to support the growth of similar centers elsewhere. Because of the paucity of epidemiological data of this patient population, it would benefit the scientific community if research were directly integrated into the business model from the initial establishment of the center.

## Conclusion

As a result of a large deficit in EDS/HSD-informed care providers and a consensus among patients describing ineffective treatment and harm done by inadequate medical care, patients with suspected or confirmed EDS, HSD, and related disorders who live in the Chicagoland area would benefit from a collaborative medical center to identify, diagnose, and treat these largely underdiagnosed conditions. Creation of such a center would offer synergistic benefits to the patients, medical providers and the healthcare facilities that serve them. Patients would have access to more streamlined, comprehensive, accessible and expert care, overburdened specialty care providers would have access to a collaborative team to share their heavy patient loads and long wait times, and facilities would benefit from a steady stream of currently underserved patients who will provide significant sources of revenue as a result of their complex medical needs. As the breadth of medical knowledge to diagnose and treat disease in the future expands, primary care providers who encounter misunderstood conditions such as these will require additional support that a collaborative center such as the one proposed would offer.

## Figures and Tables

**TABLE 1:** DEMOGRAPHICS

		N*	%
Gender	Male	16	5%
	Female	262	89%
	Transgender	3	1%
	Nonbinary	15	5%
Age	18–24	47	16%
	25–34	84	28%
	34–45	74	25%
	44–55	61	21%
	54–65	27	9%
	65 and older	3	1%
Residence	Chicago	59	22%
	Chicagoland suburbs	166	62%
	Elsewhere in Illinois	19	7%
	Elsewhere in the USA	18	7%
	Outside the United States	7	3%

* Not all respondents answered each question.

**TABLE 2:** COMORBIDITIES

	Formally diagnosed		Suspected (N, %)	
	N	%	N	%
Anxiety	179	58%	35	11%
Arthritis	108	35%	41	13%
Craniocervical instability	43	14%	103	33%
Depression	138	45%	39	13%
Dysautonomia	170	55%	70	23%
Fibromyalgia	80	26%	36	12%
Irritable bowel syndrome	135	44%	66	21%
Mast cell activation syndrome	66	21%	138	45%
Migraine	155	50%	53	17%
Temporomandibular joint dysfunction	120	39%	73	24%

**TABLE 3:** MEDICAL CARE FUNDING

		N	%
Insurance status	Medicare	13	4%
	Medicaid	30	10%
	Private	219	74%
	Combination public/private	20	7%
	None	3	1%
	Other	11	4%
Last year’s healthcare expenditure	$0–999	25	9%
	$1,000–4,999	93	32%
	$5,000–9,999	86	30%
	$10,000–19,999	62	21%
	$20,000 or more	25	9%

* Not all respondents answered each question.

## Data Availability

Material is available from the corresponding author.
